# Tailored multimodality therapy guided by a two-step decision making process for head-and-neck cancer of unknown primary

**DOI:** 10.18632/oncotarget.9492

**Published:** 2016-05-20

**Authors:** Shengjin Dou, Wei Qian, Qinghai Ji, Zhuoying Wang, Guopei Zhu

**Affiliations:** ^1^ Department of Radiation Oncology, Fudan University Shanghai Cancer Center, Shanghai 200032, China; ^2^ Department of Head and Neck Surgery, Fudan University Shanghai Cancer Center, Shanghai 200032, China; ^3^ Department of Oral and Maxillofacial-Head and Neck Oncology, Ninth People's Hospital, Shanghai Jiao Tong University School of Medicine, Shanghai, 200032, China

**Keywords:** unknown primary carcinoma, cervical laymph node metastasis, multimodality therapy, treatment decision, head and neck cancer

## Abstract

**Background:**

There is no consensus on the treatment of head-and-neck cancer of unknown primary (HNCUP). The objective of this study is to report our single institution's experience of a tailored multimodality therapy guided by a two-step decision making process.

**Materials and Methods:**

From January 2007 to November 2013, 92 consecutive patients of HNCUP were treated. 77 patients were treated according the process above, 24 were treated by radiotherapy to the nasopharyngeal site, 7 received neck dissection and radiotherapy to other putative mucosal site, 30 were treated by neck dissection alone, and 16 received neck dissection followed by radiotherapy to the neck. SPSS 20.0 software was used for statistical analysis.

**Results:**

After a median follow-up of 34 months, the 3-year overall survival rate was 84.5%. The 3-year mucosal control rate, neck control rate, distant metastasis-free survival rate and disease-free survival rate were 80.9%, 76.2%, and 92.0%, respectively. Of the 24 patients treated as putative nasopharyngeal carcinoma, no primary emerged from any site. Primary tumor emerged in 14 patients, and no primary emerged in the 31 patients treated with putative site radiation (3-year mucosal control rate: 100% vs. 67.9%, p = 0.010). Of the 46 patients treated with neck dissection with/without postoperative radiation, 14 developed neck recurrence, and patients without postoperative radiation suffered more ipsilateral neck recurrence.

**Conclusions:**

The two-step decision-making process seem to be reasonable in treating Chinese HNCUP patients. However, this results need to be prospectively validated.

## INTRODUCTION

Head-and-neck cancer of unknown primary (HNCUP) represents a heterogeneous group of cancers that account for approximately 3% of all head and neck malignancies [1]. Despite its heterogeneity, HNCUP has traditionally been treated as a single entity. However, with increased understanding of HNCUP in the past decades, the treatment strategy has evolved from empirical treatment to tailored individualized therapy for putative primary cancer based on sophisticated imaging, immunohistochemical tests, and molecular profiling tools [2].

The radiation field has also evolved from comprehensive treatment that includes the whole pharynx-axis to accurate and individualized treatment. Oropharynx-targeted radiotherapy sparing the larynx has been widely accepted in non-Asian patients [3, 4]. However, Asian patients are more likely to develop squamous cell carcinoma (SCC) of the nasopharynx, which is associated with Epstein−Barr virus (EBV), nasopharynx-targeted radiotherapy should be considered in certain HNCUP patients in China.

**Figure 1:**
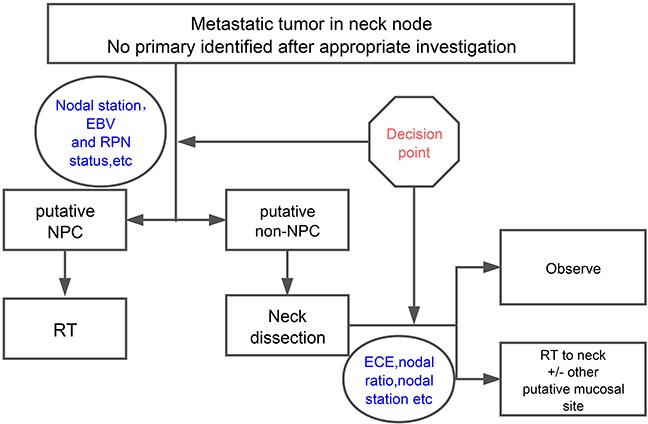
The two-step decision making process guided HNSCC multidisciplinary treatment In our center, multimodality treatment was made through a two-step decision making process. The first step was to distinguish these patients from putative nasopharyngeal origin, which would be treated mainly by radiotherapy. For other patients who were defined as non-nasopharyngeal origin, surgery was preferred. The second step was to determine whether postoperative radiotherapy or concurrent chemoradiotherapy should be given to the high risk patients of the latter group. Abbreviation: EBV: Epstein−Barr virus; RPN: retropharyngeal node; NPC: nasopharyngeal carcinoma; RT: radiotherapy; ECE: extracapsular extension.

In our institution, we manage HNCUP according to the pattern of cervical lymphatic metastasis: patients with putative nasopharyngeal origin are treated by radiotherapy, the others are treated by upfront neck dissection [5]. The treatment decision was made by a multidisciplinary team based on a two-step principle (Figure 1). The first step was to identify patients who have putative nasopharyngeal origin based on nodal station, retropharyngeal node status,EBV and suspicious abnormalities from physical and imaging examinations. These patients were treated mainly by radiotherapy. For the other patients, upfront neck dissection was preferred. The second step was to determine whether adjuvant radiotherapy/concurrent chemoradiotherapy should be given to the latter group. Postoperative radiotherapy/chemoradiotherapy to the neck and putative mucosal site other than nasopharynx was done selectively based on different risk factors (extent of neck mass, extracapsular spread, lymph node ratio etc.) The objective of this study was to report our single institution experience in long-term outcomes of patients with HNCUP treated with tailored multimodality therapy guided by this two-step decision making process.

## RESULTS

### Patients

A total of 77 patients were retrospectively enrolled. The patients' demographic and tumor characteristics are listed in Table 1.

**Table 1:** Characteristics of 77 patients

Characteristics	No
Age(years)	
Median	57
Range	31-75
Gender	
Male	64(83%)
Female	13(17%)
N status	
N1	8(10%)
N2a	16(21%)
N2b	38(49%)
N2c	13(17%)
N3	2(3%)
Lymph node involvement	
Level I	6(8%)
Level II	70(91%)
Level III	42(55%)
Level IV	17(22%)
Level V	11(14%)
Bilateral	15(20%)
ECE	
positive	37(48%)
negative	37(48%)
unknown	3(4%)
Pathology type	
SCC	46(60%)
poorly differentiated carcinoma	29(38%)
undifferentiated carcinoma	2(3%)
Grade	
High	50(65%)
Intermediate	4(5%)
Low	7(9%)
Unknown	16(21%)
EBV VCA-IgA	
Positive	20(26%)
Negative	23(30%)
unknown	34(44%)
RPN	
positive	12(16%)
negative	65(84%)
Radiation dose(Gy)	
Median	66
Range	54.0-76.6

The median age was 57 years and 83% of the patients were male. Level II lymph nodes were mostly involved (91%) followed by level III (55%), whereas level I involvement was relatively rare (8%). Only 4 (5%) patients presented with solitary level I lymph node metastasis. The clinical N classification in the majority of cases was N2b (49%). Pathology results were obtained from a fine needle biopsy in 24 cases (31%) and neck dissection in 53 cases (69%). An EB virus VCA-IgA test was done in 43 cases (56%), and approximately half of these (47%) were positive. There were 12 (16%) patients also had involvement of retropharyngeal lymph nodes, 8 of which were pathologically confirmed by transoral ultrasound-guided fine needle aspiration [6, 7]. Extracapsular extension (ECE) was diagnosed by pathological examination or imaging studies in 37 (48%) patients. Signs of ECE on imaging studies included infiltration of adjacent structures. All patients were treated with curative intent.

### Outcomes of the whole group

The median follow-up time was 34 months (range 5-91 months). The 3-year OS was 84.5%. The 3-year MC, NC, DMFS and DFS were 80.9%, 76.2%, 92.0%, and 59.4%, respectively.

**Figure 2:**
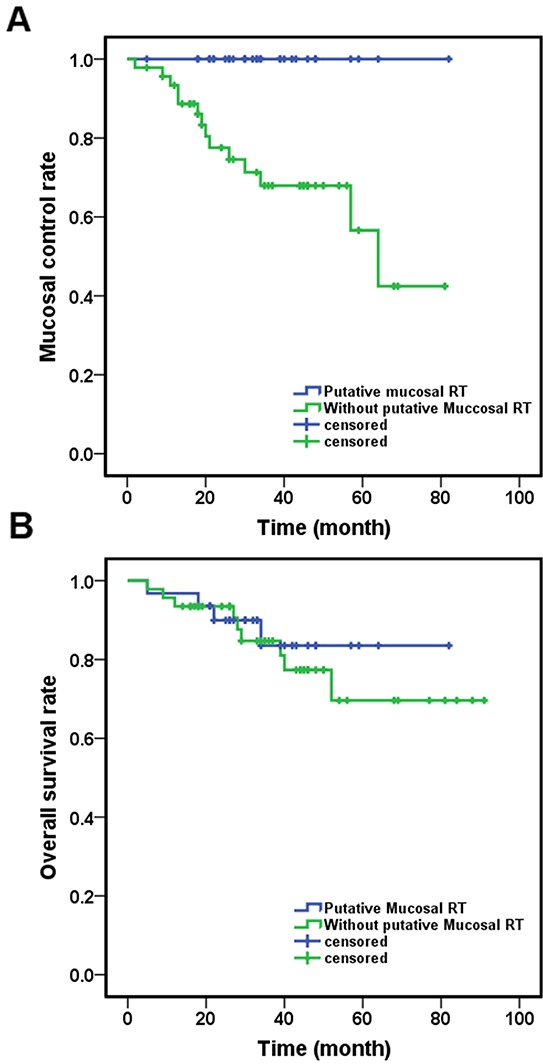

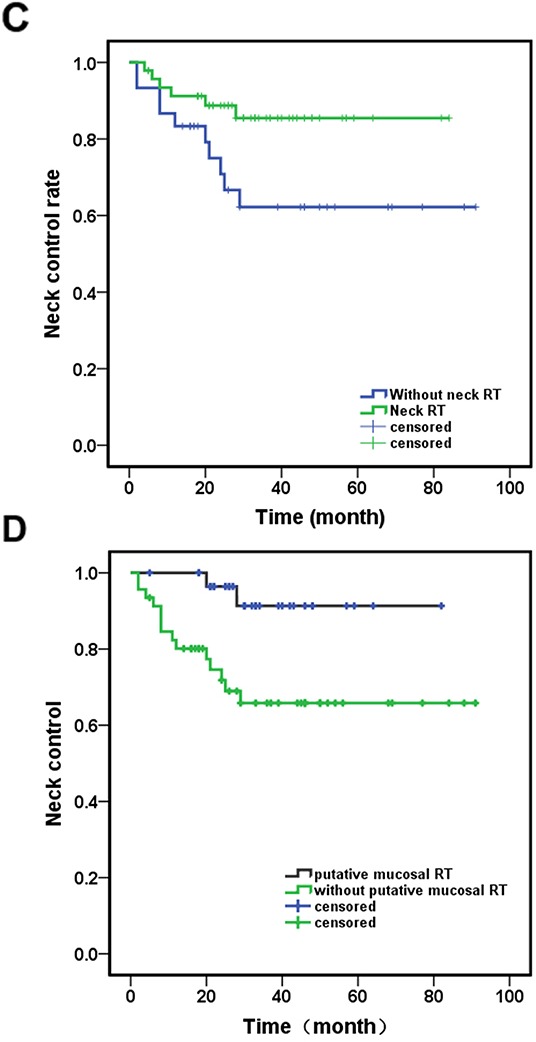
Different outcomes of the patients received different treatment **A.** Patient received putative primary mucosal irradiation had a significantly better MC (p=0.010); **B.** There were no difference in OS between patients received putative mucosal irradiation or not (p=0.591). **C.** Patients received neck irradiation have better NC (p=0.037); **D.** Patients received putative primary mucosal irradiation have better NC (p=0.010). Abbreviation: RT: radiotherapy; MC: mucosal control; OS: overall survival; NC: neck control.

Primary tumors emerged in 14 (18.1%) patients (10 patients in Group C and 4 patients in Group D). The median time to primary emergence was 19.5 month (range 2-64 month). No primary tumor emerged in patients who were treated with putative site radiation. When comparing patients treated with putative site radiation (Groups A and B) to those without (Groups C and D), the 3-year MC was 100% compared with 67.9% (p= 0.010) (Figure 2A), but no differences were observed in 3-year OS (83.5% vs 84.7%, p=0.591) (Figure 2B).

Sixteen patients developed neck recurrence, 14 of them in Groups C and D (10 in Group C and 4 in Group D). The median time to neck recurrence was 11.5 months (range 2-39 month). Two patients treated with neck dissection alone (Group C) recurred two months after surgery, both presented with a nodal mass more than 4 cm with ECE. Patients receiving neck irradiation (Groups A, B, D) had a better 3-year NC (87.5% versus 62.2%, p=0.037) (Figure 2C).

When comparing patients who received putative site radiation to those who did not, the 3-year NC of Groups A and B was 91.4%, whereas that of Groups C and D was 65.8% (p=0.010) (Figure 2D).

Six patients (3 in Group A, 2 in Group B, and 1 in Group C) developed distance metastasis between 2 and 12 months after diagnosis, 5 of them ultimately died of disease. The most common site of metastasis was bone (5 patients), followed by liver and other sites.

### Emergence of nasopharyngeal carcinoma and verification of the first step of decision

A total of 24 patients were treated with putative nasopharyngeal carcinoma (NPC). Of these, 22 patients primarily presented with level II involvement, 8 with bilateral diseases. Thirteen of them were EBV VCA-IgA positive and 11 RPN positive. The other 2 patients were RPN positive, one with a level Ib lymph node only and the other with a level III lymph node only.

Three of 6 patients with level II involvement who were both PRN and EBV negative had abnormal findings in nasopharynx in the MRI study. Four of 12 patients who had PET/CT imaging showed high FDG uptake in the nasopharynx. All had negative nasopharynx biopsies.

There were no local recurrence or other primary site emergence observed in all patients treated with putative NPC (Group A). However, 3 patients who were treated with neck dissection alone (Group C) presented with NPC during follow up. In other words, 4% of the patients failed in the first decision-making step. Of these 3 patients, 1 patients presented with level III and level IV positive lymph nodes, the other 2 patients presented with multiple lymph nodes metastasis of level II-V and positive EBV VCA-IgA test, but the nasopharyngeal MRI and PET/CT was negative.

### Verification of the second decision-making step

Fifty-three patients were assumed to be non-NPC. Seven of them were treated with putative mucosal sites radiation (Group B), the rest (46) was not (Group C and D).

Of the 11 emerged non-NPC primary tumors, 3 (21.4%) were found in the oropharynx, 3 (21.4%) in the oral cavity, 3 (21.4%) in the pharynx and hypopharynx, 1 (7.1%) in paranasal sinus, and 1 (7.1%) in the esophagus. Of the four patients presenting with solitary level I lymph node involvement, primary tumor emerged in 2 patients, one in the maxillary sinus at 13 months, the other in the gingiva at 18 month. Two patients with solitary level III involvement presented an oropharyngeal carcinoma at 11 and 34 months, respectively.

Of the 46 patients who were treated with neck dissection with (Group D) or without postoperative radiation (Group C), 14 developed neck recurrence. Four patients in Group D (4/16, 25%) developed neck recurrence; all occurred in the contralateral neck. Ten patients in Group C (10/30, 30%) had neck recurrence and 8 of them had recurrence in the ipsilateral neck, and six of them presented with nodal mass with ECE.

Five patients developed emerging primary tumors concurrently or after neck recurrence. Two patients with contralateral recurrence presented with nasopharyngeal carcinoma at the same time. There was no difference in the 3-year NC between Groups C and D (62.2% vs. 73.7%, p=0.748).

### Prognostic factors

Prognostic analysis was done for the whole group of 92 patients. The impact of the following potential prognostic factors on OS, DFS, DMFS, NC and MC were evaluated: age (≤57years vs. >57years), ECE, N stage (N1 and N2a vs. N2b and higher), pathological grade (high vs. low and intermediate), involvement of lymph node levels IV and V, RPN status and EBV VCA-IgA results. Furthermore, the influence of mucosal irradiation, neck irradiation and chemotherapy on the endpoints described above was investigated. The result of univariate analysis was showed in Table 2.

**Table 2:** Univariate analysis with 3-year NC, MC, DMFS, DFS and OS and significance of analyzed prognostic factors

Prognostic factor	N(%)	3-y NC	P value	3-y MC	P value	3-y DMFS	P value	3-y DFS	P value	3-y OS	P value
Age			0.454		0.888		0.344		0.128		**0.008**
≤57	48 (52%)	84.6%		84.2%		95.8%		73.8%		92.8%	
>57	44 (48%)	72.8%		81.1%		90.6%		52.2%		80.7%	
**N stage**			**0.024**		**0.621**		**0.939**		**0.202**		**0.706**
≤N2a	30 (33%)	93.2%		84.8%		93.3%		71.6%		85.7%	
>N2a	62 (67%)	72.1%		82.1%		93.3%		59.9%		82.1%	
Level IV,V involvement			0.143		0.684		0.648		0.368		0.789
Yes	23 (25%)	67.8%		85.2%		90.9%		60.2%		82.9%	
No	69 (75%)	83.4%		82.7%		94.2%		65.8%		88.3%	
**ECE**			**0.197**		**0.526**		**0.289**		**0.228**		0.002
Yes	41 (45%)	71.9%		86.4%		90.0%		57.2%		72.5%	
No	47 (51%)	84.7%		80.4%		95.7%		68.9%		97.9%	
Grade			0.946		**0.000**		0.311		**0.003**		0.107
High	64 (70%)	80.8%		88.4%		90.5%		70.3%		87.2%	
Other	11 (12%)	81.8%		26.9%		**100%**		13.9%		79.5%	
**EBVCA-IgA**			**0.016**		**0.097**		**0.642**		**0.010**		**0.143**
** Positive**	23 (25%)	95.7%		94.7%		95.7%		85.6%		95.5%	
** Negative**	27 (29%)	66.1%		76.5%		92.6%		49.6%		82.8%	
**RPN**			0.078		0.437		**0.006**		0.694		0.271
** Positive**	13 (14%)	**100%**		88.9%		76.9%		68.4%		76.2%	
** Negative**	78 (85%)	75.9%		82.0%		96.0%		62.9%		89.1%	
**Chemotherapy**			**0.003**		**0.016**		0.195		**0.003**		0.354
** Yes**	54 (59%)	89.2%		89.4%		96.3%		73.6%		86.5%	
** No**	38 (41%)	64.8%		73.4%		89.0%		49.7%		88.6%	
**Neck irradiation**			**0.037**		**0.002**		0.404		**0.013**		0.472
** Yes**	62 (67%)	87.5%		92.2%		91.9%		73.6%		87.0%	
** No**	30 (33%)	62.2%		64.4%		96.4%		42.8%		87.5%	
**Mucosal irradiation**			**0.022**		**0.004**		0.382		**0.005**		0.910
** Yes**	31 (34%)	91.4%		**100%**		90.3%		82.4%		83.5%	
** No**	61 (66%)	73.1%		74.9%		94.8%		54.2%		88.7%	

Based on the univariate analysis, the presence of ECE and age above 57 years old were associated with a significantly shorter OS (p = 0.002 and 0.008, respectively). These two factors remained statistically significant in the multi-variant analysis. Univariate analysis showed that chemotherapy, neck irradiation and mucosal irradiation were associated with better outcomes of NC, MC and DFS; however, no statistically significant differences were observed for 3-year DMFS and OS.

RPN status was found to be the only prognostic factor associated with poor DMFS. Although pathological grade adversely impacted MC and DFS, N stage was associated with poor NC, EBV VCA-IgA was associated with better NC and DFS, and the N stage was only associated with worse NC. Multivariate analysis revealed that chemotherapy was an independent prognostic factor for both NC (p=0.023) and DFS (p=0.001), and pathological grade was identified as a significant predictive factor for both MC (p=0.020) and DFS (p=0.025).

## DISCUSSION

The optimal treatment of HNCUP remains controversial and lacks evidence from prospective randomized trials. The management of these patients relies primarily on surgery and radiotherapy. The role of radiotherapy in sterilizing putative mucosal sites remains controversial. The main debate concerns the extent of the radiation field. Although pan-mucosal irradiation from the nasopharynx to the hypopharynx and bilateral neck nodes reduces the risk of emergence of a mucosal primary or a nodal relapse, it has been associated with significant toxicity and long-term morbidity (mostly xerostomia and dysphagia) [8]. Most single-institution retrospective studies have not shown any advantage for more extensive irradiation [9, 10]. Therefore, extensive irradiation, including the putative mucosal site might be appropriate only for selected patients.

The decision of whether to employ putative mucosal irradiation should be made based on individual patient including the potential primary site from clinical information and performance status. Mourad et al. reported their initial experience with oropharynx-targeted radiation therapy for HNCUP [3]. Sixty-eight patients received irradiation to the oropharynx, RPN, and bilateral neck, and 56% of them underwent concurrent platinum-based chemoradiotherapy. At a median follow-up of 3.5 years, the actuarial locoregional control was 95.5%. The emergence of primary tumor developed only in 1 patient (1.5%) and 2 patients (3%) failed in the neck. Long-term radiotherapy toxicity was grade 1 xerostomia (68%), dysphagia (35%), neck stiffness (15%) and trismus (6%). The authors concluded that oropharynx-targeted radiotherapy for non-Asian patients provides excellent oncological and functional outcomes.

Because Asian patients with neck lymph node metastasis are more likely to have a nasopharyngeal origin, we developed a two-step decision-making process and observed similar outcomes. The 3-year OS, MC, NC and DMFS was 84.5%, 80.9%, 76.2%, and 92.0%, respectively, which was comparable to the results obtained by Mourad et al.[3] No primary site emerged in the 31 patients receiving radiotherapy to the putative mucosal site, which may prove that the decision to irradiate the mucosal site was appropriate, and radiation may have sterilized the occult primary tumor. Moreover, we revealed that patients treated with putative primary had significant less neck recurrence (91.4% vs. 65.8%. p=0.010), which would suggest that the eradication of the primary also resulted in better regional control and indicates that we made the correct decisions in the first step. The purpose of distinguishing putative NPC as the first step is that NPC was treated mainly by radiotherapy, whereas surgery is the main treatment for other head and neck carcinomas. Thus, the right decision for the first step can limit unnecessary neck dissection. However, 3 of the 14 primary sites were still found in the nasopharynx, and we were unable to conclude putative NPC on these patients based on the clinical characteristics at that time. A more specific evaluation process including molecular assays may help to improve the first step of decision. Detection of Epstein–Barr virus (EBV) in an involved cervical lymph node or EBV DNA in the plasma/serum may suggest a nasopharyngeal origin [11, 12]. Emerging data also suggest that molecular profiling and tissue of origin assays have a place in the management of HNCUP patients [13, 14]. Because these molecular assays were not routinely tested in our institution, we were unable to validate these methods.

**Figure 3:**
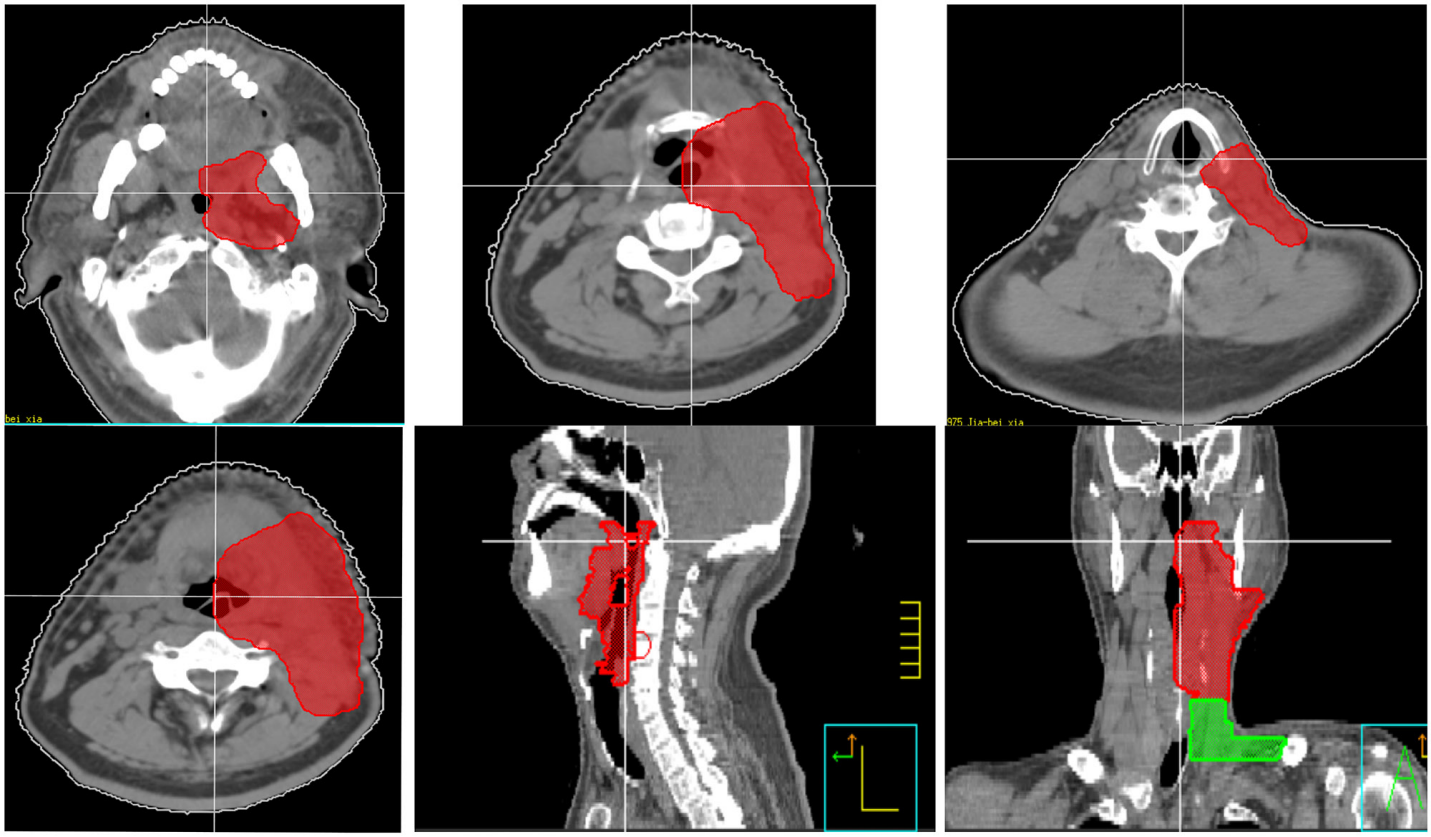
New target volume delineation standard for non-NPC patients (clinical target volume for elective mucosal irradiation) The target volume including unilateral oropharynx, hypopharynx, supraglottic structures and unilateral neck, excluding oral cavity, vocal cord and cervical esophagus. For midline structures, such as base of tongue, soft palate and epiglottis, the target volume should include part of contralateral structures.

However, as for Group C and D, our retrospective data showed that there was a relative high incidence of primary emergence (14/46,30.%) and neck recurrence (14/46, 30%), which demand us to improve our treatment for these patients. In addition to neck irradiation, a relative comprehensive mucosal site should be included. Since 2014, we designed a new target volume (clinical target volume for mucosal site) delineation standard for these patients. The target volume including unilateral oropharynx, hypopharynx, supraglottic structures and unilateral neck, excluding oral cavity, vocal cord and cervical esophagus (Figure 3). We intend to decrease the mucosal failure as well as neck failure by this radiation field coverage, and preliminary results have been achieved.

Our two-step decision making process represents a type of tailored therapy that needs multidisciplinary cooperation. Janssen et al. [15] also reported the outcomes of HNCUP cases treated with individualized IMRT. Unilateral irradiation was preferred based on individual risk factors, including clinical, surgical, histopathological, and imaging information, and the treatment fields were enlarged to the putative mucosal site or the contralateral neck. After a median follow-up of 30.5 months, the 3-year overall survival, mucosal control, neck control and distant metastasis-free survival rates were 76, 100, 93, and 88, respectively. No patient suffered from a local recurrence, and no grade II or higher late sequelae were observed. The same intention underlying tailored radiotherapy and individualized IMRT is to reduce unnecessary irradiation to innocent mucosa. However, the ideal way is to make every effort to define the primary site and thereby decrease the diagnosis of unknown primary HNSCC [16].

This study has several limitations. First was the retrospective property: EBV status was not known for about half patients; As for the second step, there was no standard protocol to treat the patient, whether to treat the putative mucosal site or whether chemotherapy should be done was decided by different physician, so the results need to be further confirmed prospectively. Second, the study population was small; however, this is difficult to avoid when dealing with such a rare entity.

## CONCLUSIONS

This tailored multimodality therapy guided by a two-step decision-making process results in high rates of OS, MC, NC, and DMFS. The two-step process seems to be reasonable in treating Chinese HNCUP patients. However, this results need be prospectively validated with a larger number of patients.

## MATERIALS AND METHODS

### Eligibility of patients

From January 2007 to November 2013, 92 consecutive HNCUP patients were treated in our institution. Fifteen patients were treated with neck irradiation alone per treating physicians not according to the decision-making process described here and were thus excluded. For the remaining 77 patients included in the analysis, the diagnosis was histologically confirmed including metastatic SCC, poorly differentiated carcinoma, or undifferentiated carcinoma. All patients underwent comprehensive workup, including complete physical examination, CT and/or MRI of the head and neck, and panendoscopy with directed biopsies that did not identify any primary site. Forty-three (46.7%) patients also had an FDG-PET/CT study at the initial evaluation. There were no patients with distant metastasis at presentation or with metastases limited to supraclavicular lymph nodes. Because there is no staging system for HNCUP, the regional lymph node staging from the American Joint Committee on Cancer (AJCC, 7th edition) staging for head and neck sites was used.

### Treatment

The treatment decision for all patients was made by a multidisciplinary team including surgeons, radiation oncologists, medical oncologists, radiologists, and pathologists. Multimodality treatment was made through a two-step decision-making process (Figure 1) based on the risk characteristics of each patients.

The treatment of these patients could be divided into following groups:

a Group A: Treated as putative NPC (24 patients);
b Group B: Neck dissection and irradiation to other putative mucosal site and bilateral neck (7 patients);
c Group C: Neck dissection alone (30 patients);
d Group D: Neck dissection followed by ipsilateral neck irradiation (16 patients);

Putative NPC was considered if the involved lymph node was in level II, especially level IIb, or positive EBV VCA-IgA, or RPN involvement or abnormal findings in imaging studies (MRI or PET/CT) but with negative nasopharynx biopsy. As for Group B, there were no clear criteria to treat the putative mucosal site, the treatment decision was made by the treating physician.

Therefore, Groups A and B received radiotherapy to the putative mucosal sites, and Groups A, B, and D received radiotherapy to the neck.

### Radiation volumes dose prescription

We used simultaneously integrated boost (SIB) IMRT [17, 18] in all patients. For patients in group A, radiation volume included nasopharyngeal region and bilateral neck. A dose of 70/66, 60 and 54 Gy was given to gross disease, the nasopharynx region and the high risk bilateral involved neck, and bilateral low neck, respectively. For patients in Group B, the radiation volume included putative mucosal regions other than the nasopharynx and ipsilateral/bilateral neck. A dose of 70/66, 60 and 54 Gy was given to gross disease, the putative mucosal region the high-risk involved neck, and the low-risk neck, respectively. For patients in Group D, the radiation volume was confined within the neck lymph node region. Ipsilateral neck was preferred, unless bilateral extension was observed. A dose of 60 and 54 Gy was given to the high-risk involved neck and the low-risk neck, respectively. The dose of each fraction was 1.8-2.2Gy.

### Surgery

A total of 53 patients received upfront neck dissection. Elective neck dissection was done in 48 patients, while comprehensive neck dissection was done in 5 patients. Eighty percent of the cases included at least level II-IV. A total of 12 patients also received suspicious site resection (Table 3). After the completion of definitive radiotherapy, salvage neck dissection was offered to patients with residual disease.

**Table 3:** Resection of suspicious site

Suspicious site resection	No
**Parotid**	4
**Tonsil**	4
**Submandibular Gland**	4
**Total**	12

### Chemotherapy

Because the role of chemotherapy in HNCUP was still not clear, there was no standard chemotherapy protocol for these patients. Chemotherapy was administrated in 54 patients based on the extent of nodal involvement, resection status, extra-nodal extension, age, and Karnofsky performance score. Most of them received platinum-based chemotherapy.

### Observation end points and statistical analysis

Each patient had follow-up appointments every three months during the first two years, every six months in years 3-5, and annually after five years. Each follow-up included a complete history and comprehensive physical examination, direct or indirect nasopharyngoscope examination and laryngoscope examination, MRI of head and neck region, chest CT and abdominal ultrasound was done every half year. PET/CT was done in selective patients. Overall survival (OS) was calculated from the first day of diagnosis to the last follow-up or the date when the patient died from any cause. Disease-free survival (DFS) was defined from the first day of diagnosis to the day of discovery of any tumor (primary site, regional, metastatic or second primary) after treatment or death from any cause. Mucosal control (MC) was measured from the first day of diagnosis to the day of discovery of mucosal primary. Neck control (NC) was measured from the first day of diagnosis to the day of discovery of any evidence of lymph node recurrence. Distant metastasis-free survival (DMFS) was calculated from the first day of diagnosis to the day of the first discovery of any distant metastasis. Patients were censored if the defined event did not occur until the cutoff date. The survival rates were calculated using the Kaplan–Meier method and univariate analysis was tested using the log-rank test. A backward stepwise Cox regression model was used for multivariate analysis to estimate the clinical characteristics and pathological profiles for OS, DFS, MC, NC and DMFS. A p value < 0.05 was considered statistically significant. The SPSS 20.0 software was used for statistical analysis.
